# Atypical Iron-Sulfur Cluster Binding, Redox Activity and Structural Properties of *Chlamydomonas reinhardtii* Glutaredoxin 2

**DOI:** 10.3390/antiox10050803

**Published:** 2021-05-19

**Authors:** Thomas Roret, Bo Zhang, Anna Moseler, Tiphaine Dhalleine, Xing-Huang Gao, Jérémy Couturier, Stéphane D. Lemaire, Claude Didierjean, Michael K. Johnson, Nicolas Rouhier

**Affiliations:** 1 Université de Lorraine, INRAE, IAM, F-54000 Nancy, France; thomas.roret@sb-roscoff.fr (T.R.); anna-maria.moseler@univ-lorraine.fr (A.M.); Tiphaine.Dhalleine@univ-lorraine.fr (T.D.); jeremy.couturier@univ-lorraine.fr (J.C.); 2 Department of Chemistry and Centre for Metalloenzyme Studies, University of Georgia, Athens, GA 30602, USA; zb.lilacwine@gmail.com (B.Z.); mkj@uga.edu (M.K.J.); 3 Department of Genetics, Case Western Reserve University, Cleveland, OH 44106, USA; work.xinghuang@gmail.com; 4 Institut de Biologie Paris-Seine, Laboratoire de Biologie Computationnelle et Quantitative, Sorbonne Université, CNRS, UMR7238, 75006 Paris, France; stephane.lemaire@sorbonne-universite.fr; 5 Institut de Biologie Physico-Chimique, Laboratoire de Biologie Moléculaire et Cellulaire des Eucaryotes, Sorbonne Université, CNRS, UMR8226, 75006 Paris, France; 6 Université de Lorraine, CNRS, CRM2, F-54000 Nancy, France; claude.didierjean@univ-lorraine.fr

**Keywords:** glutaredoxin, glutathione, glutathionylation, iron-sulfur cluster, oxidoreductase, green alga, structure

## Abstract

Glutaredoxins (GRXs) are thioredoxin superfamily members exhibiting thiol-disulfide oxidoreductase activity and/or iron-sulfur (Fe-S) cluster binding capacities. These properties are determined by specific structural factors. In this study, we examined the capacity of the class I *Chlamydomonas reinhardtii* GRX2 recombinant protein to catalyze both protein glutathionylation and deglutathionylation reactions using a redox sensitive fluorescent protein as a model protein substrate. We observed that the catalytic cysteine of the CPYC active site motif of GRX2 was sufficient for catalyzing both reactions in the presence of glutathione. Unexpectedly, spectroscopic characterization of the protein purified under anaerobiosis showed the presence of a [2Fe-2S] cluster despite having a presumably inadequate active site signature, based on past mutational analyses. The spectroscopic characterization of cysteine mutated variants together with modeling of the Fe–S cluster-bound GRX homodimer from the structure of an apo-GRX2 indicate the existence of an atypical Fe–S cluster environment and ligation mode. Overall, the results further delineate the biochemical and structural properties of conventional GRXs, pointing to the existence of multiple factors more complex than anticipated, sustaining the capacity of these proteins to bind Fe–S clusters.

## 1. Introduction

Cellular redox homeostasis is mostly controlled by oxidoreductases belonging to the thioredoxin (TRX) superfamily. These members are characterized by the presence of a TRX fold at the structural level, which consists of a central four-stranded β-sheet surrounded by three α-helices with a βαβαββα topology [1,2]. However, some of the canonical representatives in this superfamily, TRXs and glutaredoxins (GRXs), possess one or two extra α-helices at the N-terminal or C-terminal ends [3,4]. Moreover, these proteins usually share a CxxC/S motif located on the loop between β1 and α1.

Glutaredoxins are split into at least four classes with classes I and II being the most widespread [5]. The class II GRXs have known functions as iron–sulfur (Fe–S) cluster transfer proteins due to their capacity to bind a labile [2Fe-2S] cluster into homodimers using the cysteine residues of the typical CGFS active site signature of each monomer and of two glutathione molecules [6,7]. Some class I GRXs also have the capacity to bind Fe–S clusters using a comparable ligation mode, but a distinct mode of dimerization [8,9]. However, these clusters are usually more stable and the associated functions remain unclear, although it was proposed that the transition from inactive Fe–S cluster loaded forms to active apo-forms represent a regulatory mechanism in response to the presence of oxidants [7,10,11]. In apo-forms, class I GRXs catalyze the reduction of disulfides, especially those formed upon protein glutathionylation [12]. The oxidized GRXs formed after catalysis are then primarily reduced by glutathione (GSH), itself maintained in a reduced form by NADPH-dependent glutathione reductases (GRs) [12]. Additionally, reduced class I GRXs can catalyze protein glutathionylation from oxidized glutathione (GSSG) species or potentially from glutathionylated protein donors. In this case, their redox state remains unchanged after reaction. Most class II GRXs have negligible glutathione-dependent oxidoreductase activity because of specific sequence and structural features [13,14,15,16,17].

A major structural difference distinguishing class I and II GRXs is the presence of a five amino acid insertion forming a short loop just before the CxxC/S motif [7,15]. This results in differences in glutathione binding and thus in Fe–S cluster binding and stability, between members of both classes [16,17]. Introducing or removing this loop in yeast and mammalian class I or class II GRX representatives allowed, to some extent, their functional interconversion [16,17]. Removal of the loop in class II GRXs confers oxidoreductase activity to the variants, whereas introduction of the loop in class I GRXs leads to inhibition of their oxidoreductase activity. The Fe–S cluster binding and stability are also affected by the presence/removal of this loop.

Other sequence requirements allowing Fe–S cluster coordination by GRXs were identified. It seems pretty clear that all class II GRXs coordinate a [2Fe–2S] cluster and that the CGFS signature is optimal for this function [6,18]. Nevertheless, introducing a CPxS motif in the yeast class II Grx4 is still compatible with Fe–S cluster binding [19]. In class I GRXs, it is documented that the nature of the residues surrounding the catalytic or Fe–S cluster binding cysteine is an important factor. While canonical class I GRXs have a YCPYC signature, the first described Fe–S cluster binding class I GRXs were human GLRX2 and poplar GRXC1 bearing SCSYC and YCGYC signatures, respectively [8,11]. Substituting the residue at position +1 of the most N-terminal cysteine by a proline to mimic the canonical CPYC sequence was sufficient to hamper Fe–S cluster binding in these isoforms [8,20]. Inversely, substituting the prolines present in the active sites of human GLRX1 (natural TCPYC signature) or of poplar GRXC4 (YCPYC) to mimic the sequences present in the respective paralogs was sufficient to promote Fe–S cluster binding [8,20]. This demonstrated that a proline-free active site was mandatory for assembling an oxygen-stable Fe–S cluster in these proteins. Only in one case was the TCPYC motif present in *Trypanosoma brucei* GRX1 found to be compatible with the binding on an Fe-S cluster [21]. However, in the absence of structural and mutagenesis data, the coordination mode is unclear.

Additional clues arose from the comparative analysis of two plant-specific chloroplastic paralogs, GRXC5 and GRXS12, possessing WCSYC and WCSYS signatures, respectively. Only GRXC5 formed a [2Fe–2S] cluster-bound homodimer upon expression in *E. coli* [3]. A stable Fe–S cluster could be incorporated in GRXS12 either by replacing the Trp by a Tyr and or by reintroducing the second cysteine, although the latter does not participate in Fe–S cluster ligation. Hence, in addition to the +1 position, the −1 and +3 positions are important for Fe–S cluster binding in some specific cases. Overall, it seems that class I GRXs are able to bind an Fe–S cluster as long as the amino acid combination provides enough space for the non-covalent GSH binding and the Fe–S cluster coordination.

Because *Chlamydomonas reinhardtii* GRX2 is anotherclass I GRX able to bind an Fe–S cluster despite having a CPYC signature, one aim of the study was to provide spectroscopic and structural evidence supporting this observation. Second, we aimed at expanding knowledge about the oxidoreductase activity of CrGRX2 compared to already published data by analyzing the activity of mutated variants as well as by using roGFP2 as a tool to analyze both the oxidative and reductive half-reactions of the catalytic mechanism. We show that *Chlamydomonas reinhardtii* GRX2 indeed binds a [2Fe–2S] cluster upon expression in *E. coli* despite the YCPYC signature. However, this cluster exhibits a different spectroscopic signature compared to previously characterized GRXs devoid of the proline residue. In accord with its classical 3D structure, the CrGRX2 apoprotein is shown to catalyze both protein deglutathionylation as described for many proteins but also protein glutathionylation, a feature indicating a possible dual role in the control of post-translational modification of cytosolic protein cysteine residues.

## 2. Materials and Methods

### 2.1. Cloning and Construction of Mutated Variant of C. reinhardtii GRX2 by Site-Directed Mutagenesis

The coding sequence of *C. reinhardtii* GRX2 was cloned in a modified version of pET3c as described previously [22]. Mutated sequences referred to as CrGRX2 C27S, P28G, C30S, and C56S were prepared using the QuikChange Site-Directed Mutagenesis Kit (Stratagene, La Jolla, CA, USA) with two complementary mutagenic primers carrying the desired mutation (Table S1).

### 2.2. Expression and Purification of the Recombinant Proteins

For protein production, the *E. coli* BL21(DE3) strain, containing the kanamycin-resistant pSBET plasmid, was co-transformed with the different recombinant plasmids [23]. Cultures of a single colony were progressively amplified up to 2.4 L in LB medium supplemented with ampicillin and kanamycin (50 µg/mL) at 37 °C. Protein expression was induced at exponential phase by adding 100 µM isopropyl β-D-thiogalactopyranoside for 4 h at 37 °C. The cultures were then centrifuged at 4400× *g* during 15 min.

For aerobic purification, the pellets were resuspended in about 20 mL buffer composed of 50 mM phosphate pH 8.0, 300 mM NaCl, 10 mM imidazole, and the cells were conserved at −20 °C. Cell lysis was performed by sonication (3 × 1 min with intervals of 1 min), and the soluble and insoluble fractions were separated by centrifugation for 40 min at 40,000× *g*. The soluble part was loaded on a metal ion affinity chromatography (HIS-Select^®^ Nickel Affinity Gel, Sigma-Aldrich, St. Louis, MO, USA). After extensive washing, the proteins were eluted using a 50 mM phosphate pH 8.0, 300 mM NaCl, 250 mM imidazole buffer. The recombinant proteins were concentrated and dialyzed by ultrafiltration under nitrogen pressure (Amicon, YM10 membrane) and stored in a 30 mM Tris-HCl pH 8.0, 1 mM EDTA buffer at −20 °C.

For anaerobic purification, cell pellets were resuspended in buffer A (50 mM Tris-HCl pH 7.8, 0.5 M NaCl, 20 mM imidazole, 1 mM GSH) with the addition of 10 μg/mL PMSF, 15 μg/mL DNase, and 0.5 μg/mL RNase. Cells were lysed by sonication on ice in air, and centrifuged at 39,700× *g* at 4 °C for 1 h to remove the cell debris. The extract was then taken into a Vacuum Atmosphere glove box with Ar atmosphere at oxygen levels < 5 ppm and precipitated with 60% (*w/v*) of ammonium sulfate saturation. After centrifugation outside the glove box in sealed tubes, the resulting pellet containing CrGRX2 was again taken into the glove box, resuspended in minimal volume of buffer A, and loaded onto a HisTrap HP column equilibrated with buffer A. The column was then washed with 10 column volumes of buffer A before the protein of interest was eluted using a linear gradient of imidazole from 20 to 500 mM. The fractions containing apo- and holo-CrGRX2 were separately pooled and concentrated. The anaerobic purification of CrGRX2 P28G and CrGRX2 C30S variants was carried out as described above, except that the ammonium sulfate saturation step was not performed.

### 2.3. Reduction/Oxidation of roGFP2

Reaction of CrGRX2 with roGFP2 was analyzed in vitro by ratiometric time-course measurements using a fluorescence plate reader (EnSight; PerkinElmer) with excitation at 400 ± 10 nm and 480 ± 10 nm and the detection of emitted light at 520 nm with a bandwidth of 10 nm. Mixtures containing 30 mM Tris-HCl pH 8.0, 1 mM EDTA, 1 μM roGFP2, and 1 µM CrGRX2 were pipetted into the wells of a 96-well plate with a flat bottom (OptiPlate-96 F, PerkinElmer). The measurements were carried out with initially oxidized or reduced roGFP2, respectively. For the latter, the oxidized as-purified protein was reduced with 10 mM DTT for 20 min. The remaining DTT was removed by desalting spin columns according to the manufacturer’s manual (Zeba™ Spin Desalting Columns, Thermo Scientific, Waltham, MA, USA). For reduction assays with oxidized roGFP2, GSH (buffered in 30 mM Tris-HCl pH 8.0) was added at a final concentration of 2 mM. When working with GSH, a highly negative redox state of the glutathione buffer was maintained by the addition of 0.5 U GR and 100 μM NADPH. The basal background fluorescence of buffer or of buffer containing 100 μM NADPH was subtracted from fluorescence reads for all samples. For oxidation of roGFP2, 50 μM GSSG was injected into the wells. Furthermore, H_2_O_2_ and DTT were used at a final concentration of 10 mM to preset roGFP2 to the fully oxidized and fully reduced state, respectively, and determine the maximum and minimum fluorescence ratios of roGFP2 as reference values to calculate the degree of oxidation (OxD) as described previously [24].

### 2.4. Crystallization and Structure Determination

The CrGRX2 holo-form was crystallized by the microbatch-under-oil method at 4 °C. Crystals of CrGRX2 were obtained using 1 µL protein solution (8.5 and 21 mg/mL) and 1 µL mother liquor solution containing 30% (*w/v*) PEG 8000, 0.1 M sodium cacodylate pH 6.5, and 0.2 M sodium acetate. These crystals, devoid of the brown color, were flash-frozen and stored in mother liquor supplemented with 20% (*v/v*) glycerol prior to diffraction experiments. Two data collections of CrGRX2 crystals (referred to asCrGRX2^red^ and CrGRX2^SS^, see later for explanation) were carried out. CrGRX2^red^ diffraction data were collected at 1.5 Å resolution on the FIP-BM30A beamline at the ESRF Synchrotron, processed using XDS [25] and scaled and merged with Scala from CCP4 [26]. CrGRX2^SS^ diffraction data were collected at 2.4 Å resolution on a Supernova diffractometer of the x-ray diffraction facilities of the CRM2 laboratory (Nancy, France), processed, and scaled using CrysAlisPro (Agilent). The CrGRX2 crystal structure was solved by molecular replacement with human GLRX2 (pdb entry 2FLS) using Molrep [27]. Refinement was carried out using phenix.refine [28] interspersed with manual model revisions using the program Coot [29]. All statistics are summarized in Table 1. The final models were assessed using MolProbity [30].

### 2.5. Analytical and Spectroscopic Methods

Protein concentrations were determined by the DC protein assay (Bio Rad) using bovine serum albumin as a standard. Iron concentrations were determined colorimetrically with bathophenanthroline under reducing conditions after digesting proteins with KMnO_4_/HCl, as described by Fish [31]. A series of dilutions of a 1000 ppm atomic absorption iron standard were used to construct a standard curve.

UV–Visible absorption spectra were recorded using septum-sealed quartz cuvettes of 1 mm or 1 cm pathlength at room temperature using a Shimadzu UV-3101 PC scanning spectrophotometer. Circular dichroism (CD) spectra were recorded with the same cuvettes using a JASCO J-715 spectropolarimeter (Jasco, Easton, MD, USA). For low temperature resonance Raman spectra, samples were concentrated to ~2 mM in [2Fe–2S] clusters and frozen as droplets on an O-ring-sealed gold-plated copper sample holder [32] mounted to the cold finger of a Displex Model CSA-202E closed cycle refrigerator (Air Products, Allentown, PA) at 17 K. Resonance Raman spectra were acquired using a Ramanor U1000 spectrometer (Instruments SA, Edison, NJ) coupled with a Sabre argon-ion laser (Coherent, Santa Clara, CA, USA). The spectroscopic data presented in this work are representative of a single experiment, but have been repeated at least three times with the same results.

## 3. Results and Discussion

CrGRX2 is a class I GRX member that possesses a classical YCPYC signature with two cysteine residues present at positions 27 and 30, whereas a third, non-conserved, cysteine is present at position 56 (Figure S1). CrGRX2 consists of 107 residues and all residues important for glutathione binding are present. After expression in *E. coli* of the full-length protein, the cell lysate had the typical brownish color of Fe–S cluster binding proteins. The N-terminal His-tagged protein was purified aerobically by metal affinity chromatography and in the absence of GSH, which is usually known to stabilize the Fe–S cluster, to obtain the apo-form. To obtain a holo-form, the protein was purified in the presence of 1 mM GSH under anaerobiosis.

### 3.1. Fe–S Cluster Binding Properties of Chlamydomonas Reinhardtii GRX2

**Figure 1:**
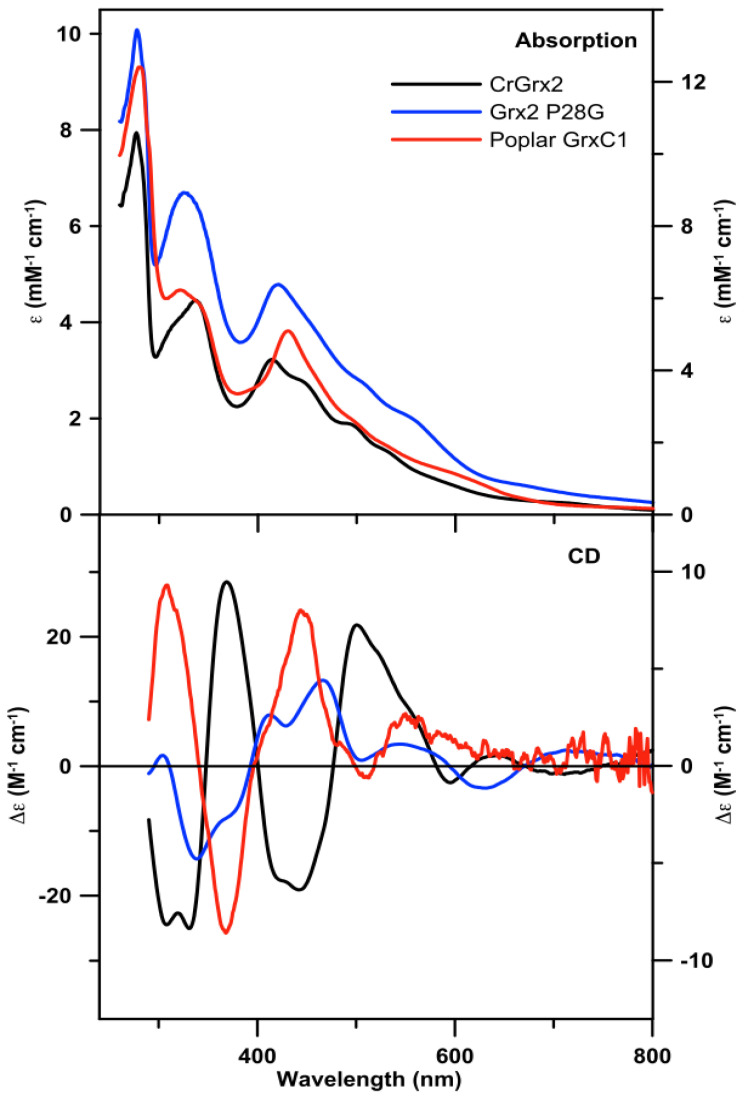
UV–Visible absorption and CD spectra of the [2Fe–2S]^2+^ cluster center in CrGRX2 (black line), CrGRX2 P28G (blue line), and poplar GRXC1 (red line). Spectra were collected in sealed 0.1 cm cuvettes under an anaerobic condition. Molar extinction coefficients (ε) values were based on the concentration of protein monomer as determined by protein assays and Δε values are based on the concentration of [2Fe–2S]^2+^ clusters.

The properties of the Fe–S center in anaerobically-purified CrGRX2 were assessed using UV–Visible absorption and CD, and resonance Raman spectroscopies. The UV–Visible absorption and CD spectra of CrGRX2 are characteristic of a [2Fe–2S]^2+^ cluster (Figure 1) [33,34]. Based on analytical measurements, the sample contained 0.52 mol of Fe per mol of protein monomer. Assuming that CrGRX2 incorporates one [2Fe–2S]^2+^ cluster per homodimer as in characterized poplar GRXC1 and human GLRX2 [8,9,11], the analytical, absorption, and CD data indicated that only 52% of anaerobically-purified CrGRX2 was occupied with the [2Fe–2S]^2+^ cluster. 

**Figure 2:**
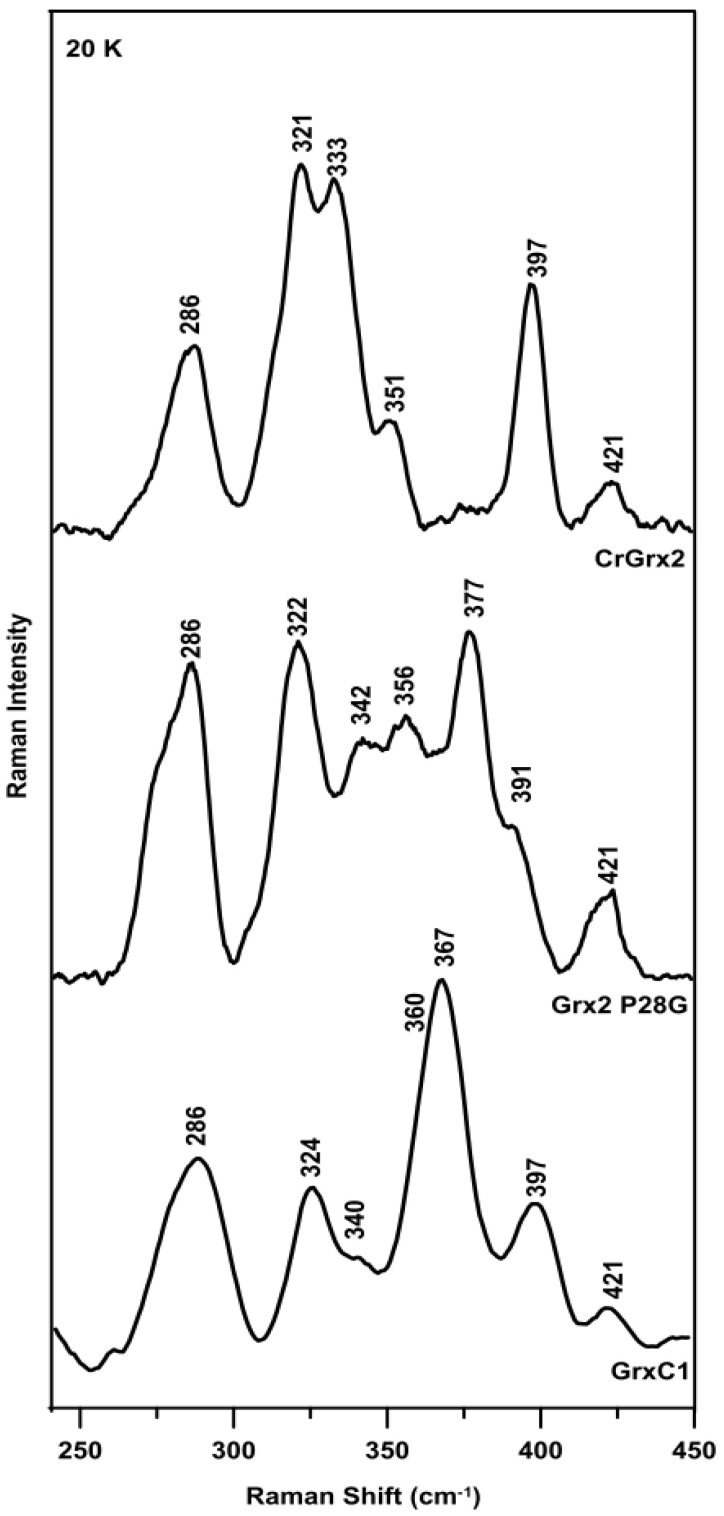
Resonance Raman spectra of purified [2Fe–2S]^2+^ cluster-bound form of CrGRX2 (top panel), CrGRX2 P28G (middle panel), and poplar GRXC1 (bottom panel). Spectra were obtained at 20 K with 457.9 nm laser excitation with ~140 mW laser power at the sample. Bands due to lattice modes of ice from frozen buffer in the sample were subtracted from all spectra.

The vibrational properties of the [2Fe–2S]^2+^ cluster in CrGRX2 were characterized by resonance Raman studies in the Fe–S stretching region. The resonance Raman spectrum of CrGRX2 obtained with thee 457.9 nm excitation revealed Fe–S stretching frequencies similar to those reported for [2Fe–2S]^2+^ clusters with complete cysteinyl ligation (Figure 2) [35,36].

Based on previous observations that the positions +1 and +3 relative to the known ligating Cys residue are important for Fe–S cluster binding, we expressed two variants, one in which Pro28 is substituted by a glycine as found in plant GRXC1 isoforms and one in which Cys30 is substituted by a Ser as found in some class I GRXs. Both variants were purified under anaerobic conditions in the presence of 1 mM GSH. CrGRX2 C30S was purified devoid of the Fe–S cluster, as evidenced by UV–Visible absorption and protein and Fe analysis. In contrast, the UV–Visible absorption and CD spectra of CrGRX2 P28G were also characteristic of a [2Fe–2S]^2+^ cluster and similar to that of CrGRX2 (Figure 1). Based on protein and Fe assays, anaerobically-purified CrGRX2 P28G contained 0.77 mol of Fe per mol of protein monomer. Assuming that holo-CrGRX2 P28G also forms a homodimer, these data indicated 77% [2Fe–2S]^2+^ cluster occupancy for anaerobically-purified CrGRX2 P28G. The vibrational properties of the [2Fe–2S]^2+^ cluster in CrGRX2 P28G were also characterized by resonance Raman studies (Figure 2). The resonance Raman spectrum revealed Fe–S stretching frequencies similar to those of [2Fe–2S]^2+^ clusters with complete cysteinyl ligation. However, while the absorption, CD, and resonance Raman data all indicate the ability of the proteins to incorporate a [2Fe–2S]^2+^ cluster, there were some significant differences comparing the [2Fe–2S]^2+^ clusters in CrGRX2, CrGRX2 P28G, and poplar GRXC1. First, the UV–Visible absorption spectra of CrGRX2 and CrGRX2 P28G both comprised resolved bands centered near 330 and 420 nm and broad absorption features in the 500–600 nm region, whereas the absorption spectrum of poplar GRXC1 was dominated by a single band centered at 430 nm. The distinct features in the CD spectra for each protein also suggested differences in the protein conformation in the vicinity of the [2Fe–2S]^2+^ cluster in CrGRX2, CrGRX2 P28G, and poplar GRXC1. Notably, the CD spectrum of CrGRX2 was similar to that of poplar GRXC1 in terms of the frequencies of the observed S-to-Fe charge transfer bands, but was essentially the mirror image, indicating reversed chirality for the [2Fe–2S]^2+^ center in CrGRX2 compared to poplar GRXC1 (see Figure 1). This most likely results from changes in the positioning of α-helices in the vicinity of the [2Fe–2S]^2+^ clusters, and indicates significant secondary and/or ternary structural differences for the cluster-bound forms of CrGRX2 and poplar GRXC1. These structural differences are also manifest in the relative intensities and frequencies of the Fe–S stretching modes observed in the resonance Raman spectra of these three proteins (see Figure 2). The differences likely reflect structural changes involving bridging and terminal Fe–S bond strengths, resulting from changes in H-bonding to the S atoms from nearby donor groups in the protein or in the Fe–S–C–C dihedral angles, which could induce mixing of terminal Fe–S stretches with the S–C–C bonding coordinate [35].

### 3.2. Chlamydomonas Reinhardtii GRX2 Catalyzes Both Protein Glutathionylation and Deglutathionylation

**Figure 3:**
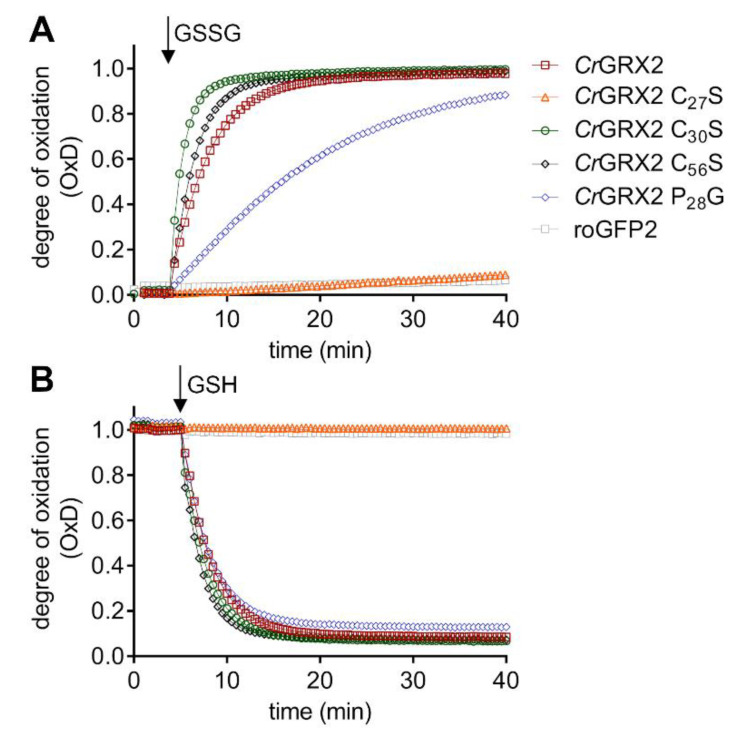
Oxidoreductase activity of CrGRX2 and its variants. Time course for the GRX-mediated oxidation (**A**) and reduction of roGFP2 (**B**) triggered by addition of 50 μM GSSG or 2 mM GSH (arrow). GSSG-dependent oxidation and GSH-dependent reduction were measured with 1 µM roGFP2 in the presence of pre-reduced 1 µM GRX2 or its variants. roGFP2 alone served as a negative control. The results shown are representative experiments obtained from 4–6 replicates.

Glutathionylation is a reversible post-translational modification consisting of the formation of a mixed disulfide between glutathione and a protein cysteine residue. Protein glutathionylation can, on the one hand, protect cysteine residues from irreversible oxidation, and on the other hand, modulate the enzyme activity [12]. It is often reported that GRXs control this modification by catalyzing protein deglutathionylation, but protein glutathionylation is described rather as a non-catalytic process occurring notably in response to stress conditions, although some examples are known where GRXs act as catalysts of protein glutathionylation [37,38,39]. Previously, we investigated the enzymatic activity profile of CrGRX2 using conventional artificial substrates (standard HED assay) prone to deglutathionylation and glutathionylated protein substrates delineating thereby CrGRX2 as an efficient catalyst of deglutathionylation [22]. Here, we used several CrGRX2 variants and the redox-sensitive green fluorescent protein 2 (roGFP2) to analyze both the reductive and oxidative half-reactions that occur through the reversible steps of glutathionylation before the formation of an intra-molecular disulfide [16]. In the presence of GSH or GSSG, respectively, we found that CrGRX2 can efficiently reduce and oxidize roGFP2 as it was shown before for several class I GRXs from other organisms (Figure 3) [15,16,17,38]. Mutating the non-conserved Cys56 into Ser did not affect the catalytic efficiency for roGFP2 reduction or oxidation. In contrast, no oxidation or reduction of roGFP2 was observed with the catalytic cysteine variant (CrGRX2 C27S), indicating an inactive protein. The variant of the second, so-called resolving, cysteine (CrGRX2 C30S), with a monothiol CPYS active site, can reduce and oxidize roGFP2 even more efficiently than the intact enzyme, suggesting that absence of the resolving cysteine does not compromise the reaction mechanism of this class I GRX. These data are in line with the ability of two different yeast monothiol class I GRXs, ScGRX7 and ScGRX2 C64S, with a natural and an engineered CPYS active-site signature, to mediate rapid oxidation and reduction of roGFP2 inside living cells and thus, to sense the glutathione redox potential in the yeast cytosol [17,40]. Furthermore, these yeast GRXs are also able to maintain the viability of yeast strains lacking all endogenous cytosolic TRXs and dithiol class I GRXs (*Δtrx1Δtrx2Δgrx1Δgrx2*) under normal growth conditions, indicating that the resolving Cys is globally dispensable [40]. The exact function and influence on the activity of the resolving cysteine remains an open question since its mutation can have different effects depending on the GRXs. Using poplar proteins, we observed that the substitution of the second Cys to a Ser leads to an increased activity of GRXC1 and GRXC2, but no real change in the case of GRXC3 and GRXC4 [41]. Together with their other redox properties, this led us to split these GRXs into two subgroups. Regarding yeast GRX1 and GRX2, both containing a CPYC motif, the substitution of the second Cys to a Ser leads to an increased activity of GRX1 and a decreased activity of GRX2 [42]. Human GLRX2 C40S exhibits increased specific activity in the HED assay [43], whereas mutating the resolving Cys of *E. coli* GRX1 decreased the activity [44]. For *C. reinhardtii* GRX1, the C58S variant catalyzes protein deglutathionylation much more efficiently than CrGRX1, but is also inactivated by H_2_O_2_ while CrGRX1 is not [45]. These results are consistent with the results obtained here with CrGRX2 C30S and with the hypothesis that under oxidizing conditions, the catalytic and resolving Cys form an intramolecular disulfide, thereby representing a protective mechanism against overoxidation [41].

For CrGRX2 P28G, which harbors a CGYC active site, we found no effect on the reduction of roGFP2, but the oxidation rate was strongly decreased. Oxidation of roGFP2 occurs in three reversible steps including the formation of the GRX-S-SG mixed disulfide, generation of the roGFP2-SG intermediate, and the subsequent formation of the intramolecular disulfide in roGFP2 [16]. Decreased activity in the oxidation of roGFP2 might result from a decreased reactivity of the CrGRX2 P28G variant with GSSG that would impact the rate of formation of the GRX-S-SG mixed disulfide. In the case of human GLRX1 and GLRX2, the GLRX1 P23S (CSYC) and GLRX2 S38P (CPYC) variants showed that the absence of the proline residue decreased the activity of the GRXs, but increased their affinity for glutathionylated substrates [43]. For the reduction of roGFP2, the GRX-S-SG intermediate reacts with a second molecule of GSH. This requires a second GSH binding site that does not include the proline residue [15,17]. Therefore, the P28G substitution is not expected to impact this second step and should have no or minor effects on the reduction of roGFP2, as consistently observed here for CrGRX2 P28G (Figure 3). In contrast, the decrease in the rate of roGFP2 oxidation is consistent with a decreased reactivity of the P28G variant with GSSG.

### 3.3. The Tridimensional Structure of Chlamydomonas reinhardtii GRX2

**Figure 4:**
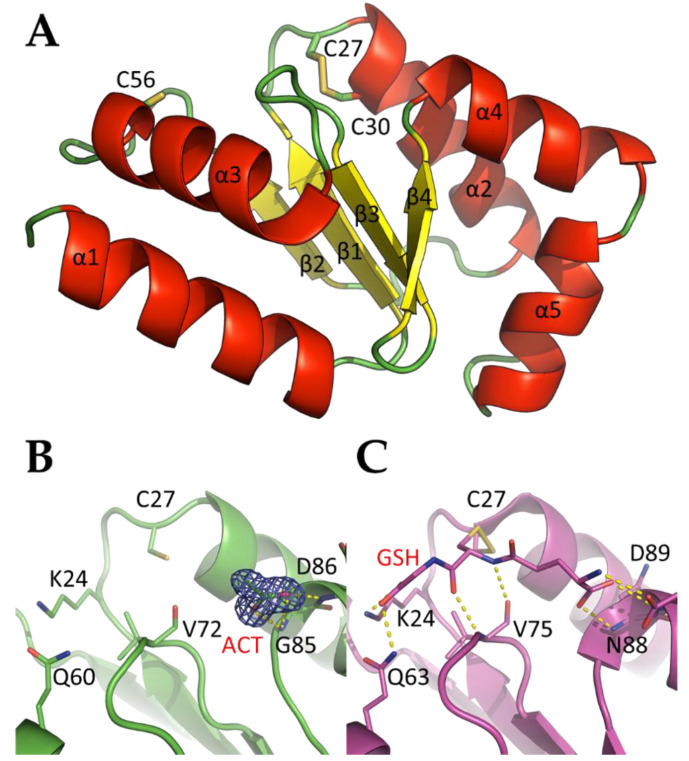
Crystal structure of *Chlamydomonas reinhardtii* GRX2. (**A**) Ribbon illustration of CrGRX2^SS^ with its cysteine residues depicted as stick models. (**B**) σA-weighted 2mFo-DFc electron density map around the acetate (ACT) molecule in CrGRX2^red^. The ACT molecule and Lys24, Cys27, Gln60, Val72, Gly85, and Asp86 residues are shown as sticks. (**C**) Interactions between the glutathione molecule and *Saccharomyces cerevisiae* Grx1 (pdb entry 3c1s). The GSH molecule and Lys24, Cys27, Gln63, Val75, Asn88, and Asp89 residues are shown as sticks.

The crystal structure of CrGRX2 was solved at a high-resolution using synchrotron radiation (1.5 Å, CrGRX2^red^) while a lower resolution was obtained using a laboratory source (2.4 Å, CrGRX2^SS^). Both crystals were isomorphous and the structures were highly similar (root-mean-square deviation (r.m.s.d.) of 0.10 Å for the superimposition of 107 pairs of Cα atoms). Only one difference was noted: Cys27 and Cys30 form a disulfide bond in the CrGRX2^SS^ structure while they were reduced in CrGRX2^red^. This point is discussed below and the high-resolution structure was used in the structural analysis. CrGRX2 crystallized in space group *P*3_2_21 (Table 1) with a single molecule of CrGRX2 (Met1-Leu107 residues) per asymmetric unit. The solvent content of the crystals was calculated to be 56%. While CrGRX2 holo-forms were used for crystallographic trials, the CrGRX2 x-ray structure is devoid of an Fe–S cluster, indicating that this latter was not stable enough in aerobic conditions. The overall fold of CrGRX2 (Figure 4A) showed four mixed β-strands in a central core structure surrounded by five α-helices. The four-stranded β-sheet is sandwiched by α-helices α1 and α3 on one side and α-helices α2, α4, and α5 on the other side. The crystal structure of CrGRX2 resembles those of the previously reported class I GRX structures (mean r.m.s.d. of 1.51 Å for the superimposition of 16 structures of class I GRXs; Figures S1 and S2).

We found an acetate molecule (Figure 4B) originating from the crystallization medium at the position usually occupied by the carboxylic acid group of the γ-glutamyl moiety of GSH (Figure 4C). The amine groups of Gly85 and Asp86, located just after two conserved glycine residues forming a structural kink, help stabilizing the acetate ion through hydrogen bonding. These positions are those usually found in the stabilization of the γ-glutamyl moiety of GSH [46]. 

**Figure 5:**
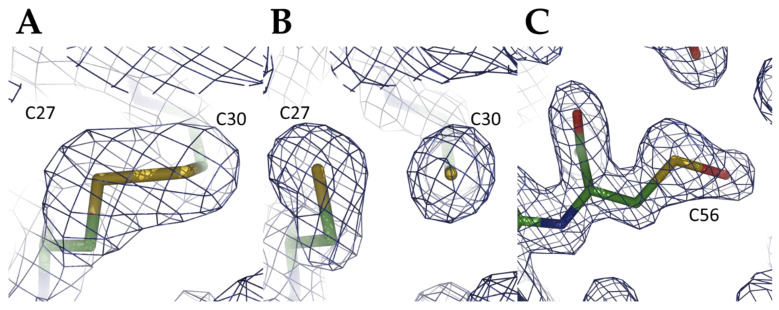
Different oxidation states of Cys27, Cys30, and Cys56 residues in the *Chlamydomonas reinhardtii* GRX2 x-ray structures. Electron-density maps displayed around (**A**) the Cys27-Cys30 disulfide bridge in CrGRX2^SS^, (**B**) the active site disulfide broken by synchrotron radiation in CrGRX2^red^, and (**C**) the Cys56-sulfenic acid in CrGRX2^red^. In each case, maps are σA-weighted 2mFo-DFc electron density maps contoured at 1.2σ around the cysteine residues. Cys27, Cys30, and Cys56 residues are shown as sticks.

The three cysteine residues (Cys27, Cys30, and Cys56) are located on the same solvent-exposed face of CrGRX2 (Figure 4A). The catalytic cysteine residue Cys27 is classically found at the N-terminal extremity of α2 and is at roughly 11 Å from Cys56 (located in α3). A disulfide bridge between Cys27 and Cys30 was observed in CrGRX2^SS^ (Figure 5A) with a distance between the Sγ-atoms of 2.1 Å. The equivalent distance of 3.2 Å in the CrGRX2^red^ structure indicates that the disulfide bond was most likely broken during the synchrotron data collection (Figure 5B). In addition to the disulfide bridge formed between the active site cysteines of CrGRX2, an extended electron density was observed in the side chain of Cys56 and was modeled as a sulfenic acid form, RS–OH (Figure 5C). The electron density near the Sγ atom of Cys56 cannot correspond to a water molecule because the distance observed between the Sγ atom and the oxygen atom was 1.7 Å. 

Interestingly, this kind of oxidation has already been observed in another class I GRX, GRX2 from *Clostridium oremlandii* (pdb entry 4TR0) [47]. While Cys56 seems to make no contribution to the oxidoreductase activity (Figure 3), its solvent-exposed position and sensitivity to oxidation may be important for regulatory purposes. For instance, human GLRX1 possesses three additional cysteines that are prone to oxidation and which were suggested to affect both the activity and the structure of the enzyme [48]. In the same superfamily, glutathionylation of an extra active site cysteine in *A. thaliana* TRX f1 prevents its recycling by ferredoxin-thioredoxin reductase [49].

After *Trypanosoma brucei* GRX1 [21], CrGRX2 is the second class I GRX with a CPYC signature shown to bind an Fe–S cluster. Former analyses suggested that the proline in the +1 position prevented Fe–S cluster coordination in GRXs [8,20]. The results obtained with *T. brucei* GRX1 and *C. reinhardtii* GRX2 suggest that some GRXs with a CPYC active site have the ability to bind the Fe–S cluster. CrGRX2 does not seem to exhibit any peculiarities at the sequence or structural level compared to other class I GRXs. Thus far, the only available models for holo-GRXs have been obtained for class I and class II GRXs devoid of proline residue in their active site signature [3,8,50,51]. Although class I and class II GRX holo-forms present distinct dimers, only local conformational changes with slight loop movements occur upon the Fe–S cluster binding in both cases. The structure of CrGRX2 is similar to other class I GRXs where the main chains of residues forming the CPYC signature have a unique conformation. Based on known crystal structures of class I holo-GRXs, we tentatively built a holo-CrGRX2. This resulted in steric clashes caused by seven residues (Lys24, Tyr26, Pro28, Tyr29, Lys32, Ser71, and Val72) (Table S2). All could be solved by modifying the rotamers of the lateral chains except for Pro28, which is too bulky to allow for the accommodation of both the glutathione molecule and the Fe–S cluster. Hence, the incorporation of an Fe–S cluster into CrGRX2 (and more generally in CPYC-containing GRXs) should be accompanied by significant structural changes, in agreement with the spectroscopic data, and rely on a new mode of dimerization. Alternatively, the second cysteine residue could act as an Fe–S cluster ligand instead of glutathione. This proposal is supported by the complete absence of an Fe–S cluster when the GRX2 C30S variant was purified under strictly anaerobic conditions as judged by UV–Visible absorption and CD spectroscopy. This is clearly in contrast with the poplar GRXC1 C34S and human GLRX2 C40S variants, which retain the capacity to bind a [2Fe–2S] cluster with the same spectral properties as the non-mutated proteins, although Fe–S cluster ligation is destabilized in the case of the poplar GRXC1 C34S variant [8,20].

## 4. Conclusions

In conclusion, using the roGFP2 as a model protein substrate, we showed that CrGRX2 catalyzes both protein glutathionylation and deglutathionylation reactions in the presence of GSSG and GSH, respectively. Both reactions require only one catalytic cysteine. Unlike deglutathionylation, the efficiency of the glutathionylation reaction is likely influenced by the nature of the residues, forming the active site signature. In addition, this work demonstrates that *C. reinhardtii* GRX2 has the capacity to bind a [2Fe–2S] cluster despite having the canonical CPYC active site signature that was shown to prevent Fe–S cluster binding in some other GRXs. While we could not obtain a crystallographic structure of the holo-form, spectroscopic evidence coupled to mutagenesis data and molecular modeling point to the existence of a new Fe–S cluster ligation mode. Whether and how this influences the Fe–S cluster associated function(s) of GRX2 will be the next question to answer. Overall, comparing GRXs from various sources again highlights the important diversity existing in terms of capacity to bind the Fe–S cluster and of oxidoreductase properties, thus allowing GRXs to play specific roles in a multitude of pathways and processes.

It is noticeable that several other proteins belonging to the TRX superfamily and with variable active site signatures have the capacity to bind an Fe–S cluster, either as isolated from *E. coli* cells or after Fe–S cluster reconstitution experiments. This is the case of an atypical protein disulfide isomerase (PDI-A, WCKHC) from plants [52,53], of mitochondrial Arabidopsis TRX o1 and o2 (WCGPC). Another interesting observation is that a single mutation is sometimes sufficient to introduce an Fe–S cluster into TRX superfamily members as exemplified by the mutation of the cis-proline at position 75 in human TRX1 and by the mutation of the TxxC active site in human PRX1 to a CxxC motif [54] or by changing the regular CGPC signature present in *E. coli* Trx1 into a CxCx motif [55]. Altogether, these observations indicate that several TRX superfamily members, not only glutaredoxins, have the capacity to bind Fe–S clusters, despite their differences both in terms of primary sequences and structural properties. This suggests that the Fe–S cluster binding capacity represent either an ancestral property present at the origin of life when the atmosphere was rich in iron and sulfur atoms, or a newly acquired property in some specific clades or organisms, which in any case led to neo- and sub-functionalization among TRX superfamily members.

## Figures and Tables

**Table 1:** Crystal structure data collection and refinement statistics. * Values in parentheses are for the highest-resolution shell.

	CrGRX2^red^	CrGRX2^SS^
**Data collection**		
Space group	*P*3_2_21	*P*3_2_21
Cell dimensions (Å)		
a, c (Å)	45.63; 104.65	45.38; 104.24
Resolution (Å)	39.53–1.50 (1.58–1.50) *	14.70–2.40 (2.53–2.40) *
No. unique reflections	20,186 (2951) *	5212 (723) *
Redundancy	3.7 (3.6) *	6.0 (3.4) *
Completeness (%)	96.5 (97.1) *	99.1 (97.1) *
*R_merge_*	0.043 (0.356) *	0.108 (0.378) *
*R_meas_*	0.049 (0.414) *	0.118 (0.435) *
*R_pim_*	0.023 (0.202) *	0.046 (0.206) *
***I***/σ***I***	12.7 (2.2) *	12.3 (3.0) *
**Refinement**		
Resolution (Å)	39.53–1.50	14.30–2.40
*R_work_/R_free_*	15.28/17.99	19.72/22.94
No. atoms		
Protein	814	794
Ligand/ion	6	4
Water	153	92
*B-factors* (Å^2^)		
Protein	24.72	24.37
Ligand/ion	26.07	23.64
Water	38.83	25.89
*R.m.s* deviations		
Bond lengths (Å)	0.015	0.002
Bond angles (°)	1.369	0.421
PDB entry	7NCV	7NCW

## Data Availability

Data regarding the 3D structures of the proteins can be found in the protein data bank under the pdb codes 7NCV and 7NCW.

## Supplementary Materials

The following are available online at https://www.mdpi.com/article/10.3390/antiox10050803/s1, Figure S1: Structural alignment of class I GRXs, Figure S2: Structural superposition of class I GRXs, Table S1: Primers used for site-directed mutagenesis experiments, Table S2: CrGRX2 residues involved in steric clashes upon dimerization around a [2Fe–2S]-cluster compared with already known class I GRX holo-forms.
