# Supplementary material for: Atypical Iron-Sulfur Cluster Binding, Redox Activity and Structural Properties of Chlamydomonas reinhardtii Glutaredoxin 2
Source: Antioxidants (Basel). 2021 May 19;10(5):803. doi: 10.3390/antiox10050803 (PMC8161271; doi:10.3390/antiox10050803)
Supplement: Supplementary file 1 [file antioxidants-10-00803-s001.zip › antioxidants-1199340-SI.pdf]

Supplementary materials associated with the paper "Atypical iron-sulfur cluster binding, redox activity and structural properties of *Chlamydomonas reinhardtii* glutaredoxin 2" by

Thomas Roret[1a], Bo Zhang[2], Anna Moseler[1], Tiphaine Dhalleine[1], Xing-Huang Gao[3], Jérémy Couturier[1], Stéphane D. Lemaire[4,5], Claude Didierjean[6], Michael K. Johnson[2], Nicolas Rouhier[1*]

Table S1. Primers used for site-directed mutagenesis experiments.

The mutagenic codons are in bold.

**Name**	**Sequence (5′-3′)**
CrGRX2 C27S for	TACAGCAAAACGTACAGCCCCTACTGCGTCAAG
CrGRX2 C27S rev	CTTGACGCAGTAGGGGCTGTACGTTTTGCTGTA
CrGRX2 P28G for	AGCAAAACGTACTGCGGCTACTGCGTCAAGGCC
CrGRX2 P28G rev	GGCCTTGACGCAGTAGCCGCAGTACGTTTTGCT
CrGRX2 C30S for	ACGTACTGCCCCTACAGCGTCAAGGCCAAGAAT
CrGRX2 C30S rev	ATTCTTGGCCTTGACGCTGTAGGGGCAGTACGT
CrGRX2 C56S for	GAGAACCGCGCCGACAGCGACGCCATGCAGGAC
CrGRX2 C56S rev	GTCCTGCATGGCGTCGCTGTCGGCGCGGTTCTC

Table S2: CrGRX2 residues involved in steric clashes upon dimerization around a [2Fe-2S]-cluster as compared with already known class I GRX holoforms.

C. reinhardtii GRX2	apo	Lys24 (*mttt*)	Tyr26 (*t80*)	Pro28 (*Cg_endo*)	Tyr29 (*m-80*)	Lys32 (*ttmt*)	Ser71 (*p*)	Val72 (*m*)
Human GLRX2	apo	Lys34 (*mttt*)	Ser36 (*p*)	Ser38 (*p*)	Tyr39 (*m-80*)	Met42 (?)	Thr80 (*p*)	Val81 (*m*)
	holo	Lys34 (*mttt*)	Ser36 (*p*)	Ser38 (*m*)	Tyr39 (*m-10*)	Met42 (*mtp/mmp*)	Thr80 (*p*)	Val81 (*m*)
A. thaliana GRXC5	apo	Lys26 (*mttt*)	Trp28 (*p-90*)	Ser30 (*p*)	Tyr31 (*m-80*)	Glu34 (*tt0*)	Thr73 (*p*)	Val74 (*m*)
	holo	Lys26 (*mttt*)	Trp28 (*p90*)	Ser30 (*m*)	Tyr31 (*m-10*)	Glu34 (*tt0*)	Thr73 (*p*)	Val74 (*m*)
Poplar GRXC1	apo	Lys27 (*mttt*)	Tyr29 (*p90*)	Gly31	Tyr32 (*m-80*)	Arg35 (*ttm170/ttm110*)	Thr73 (*p*)	Val74 (*m*)
	holo	Lys27 (*mttt*)	Tyr29 (*p90*)	Gly31	Tyr32 (*m-10*)	Arg35 (*ttm170/tpt170*)	Thr73 (*p*)	Val74 (*m*)
S. cerevisiae GRX6	apo	Lys133 (*mttt*)	Thr135 (*p*)	Ser137 (*p*)	Tyr138 (*m-80*)	Gly141	Thr182 (*p*)	Val183 (*m*)
	holo	Lys133 (*mttt*)	Thr135 (*p*)	Ser137 (*m*)	Tyr138 (*m-10*)	Gly141	Thr182 (*p*)	Val183 (*m*)

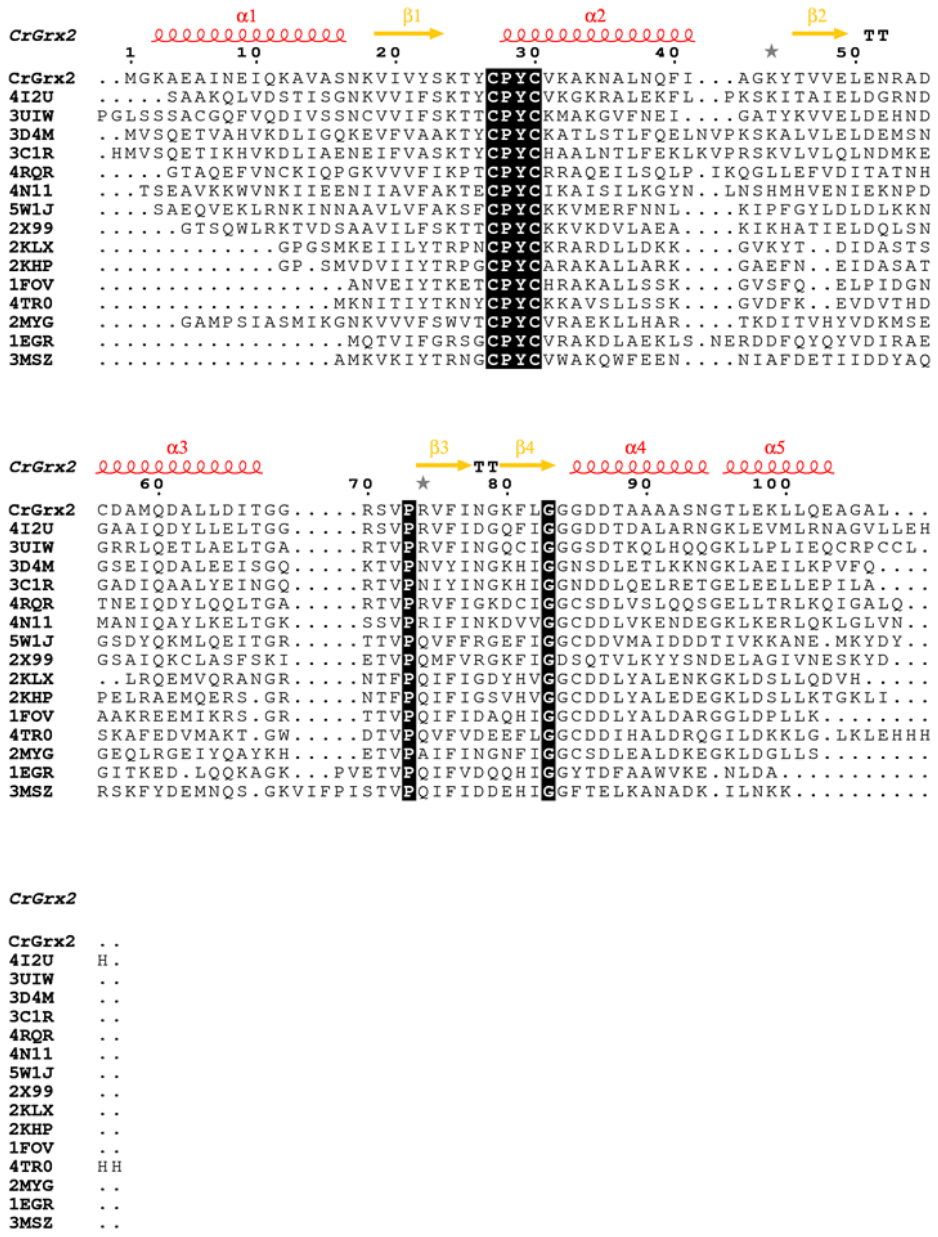

Figure S1: Structural alignment of class I GRXs.

Elements of secondary structure depicted above the sequences are based on CrGRX2 X-ray structure. Conserved residues are highlighted in black including the [27]CPYC[30] active site motif, Pro73 of the TVP-motif and Gly83 of the GG-kink. The structures used in the alignment are GRXs from *Escherichia coli* (pdb entries 1EGR and 1FOV), *Saccharomyces cerevisiae* (3C1R and 3D4M), *Danio rerio* (3UIW), *Brucella melitensis* (2KHP), *Bartonella henselae* (2KLX), *Trypanosoma brucei* (2MYG), *Francisella tularensis* (3MSZ), *Chlorella sorokiniana* (4I2U), *Plasmodium falciparum* (4N11), human (4RQR) and *Clostridium oremlandii* (4TR0) plus the GRX domains of two thioredoxin glutathione reductases from *Schistosoma mansoni* and *Echinococcus granulosus* (2X99 and 5W1J, respectively).

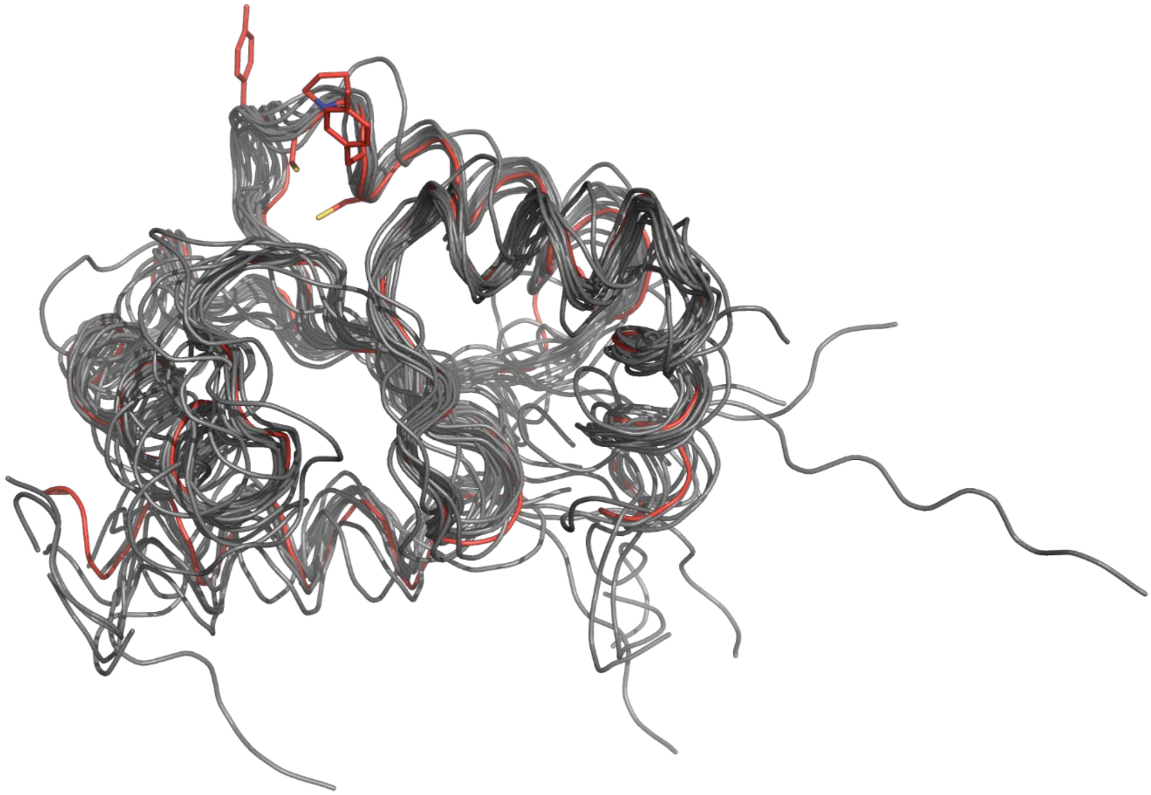

Figure S2: Structural superposition of class I GRXs.

Ribbon diagram of the superposition of CrGRX2 (red) with other class I GRX structures (grey). For clarity, only residues of the YCPYC signature are shown as sticks for CrGRX2. GRX structures used in the superposition are the same as those used in Figure S1.
